# Factors associated with orthodontist's decision to indicated orthodontic camouflage or orthognathic surgery in skeletal class III malocclusions without growth

**DOI:** 10.4317/jced.64160

**Published:** 2026-08-28

**Authors:** Claudia Carranza-Buenaño, Mariano Ortiz-Pizarro, Marcos Jimmy Carruitero-Honores

**Affiliations:** 1 Facultad de Medicina, Universidad Católica Santo Toribio de Mogrovejo, Chiclayo, Peru; 2 Facultad de Medicina, Universidad Privada Antenor Orrego, Trujillo, Peru

**Keywords:** Orthodontic camouflage, orthognathic surgery, class III malocclusion, orthodontists.

## Abstract

**Background:**

The therapeutic decision depends on a complex set of factors related to both the patient and the professional.

**Aim:**

To determine the factors associated with the orthodontist's decision to prescribe orthodontic camouflage or orthognathic surgery in skeletal Class III malocclusions without growth.

**Materials and Methods:**

An online survey was conducted with 271 orthodontists. The validity and reliability of a 16-question questionnaire were determined, and submission links were generated using SurveyMonkey software. The instrument was distributed via social media and email, achieving an 80% response rate. Pearson's chi-squared test and binomial logistic regression was used.

**Results:**

All factors evaluated were considered important in the treatment decision (p<0.001). The gonial angle was a significant risk factor (OR:5.36; 95%CI:1.53–18.72; p=0.009), as was anterior crossbite (OR:49.14; 95%CI:2.61–92.59; p=0.009). Occlusal stability (OR:0.01; 95%CI:0.00-0.29; p=0.009), third molar position and space (OR:0.14; 95%CI: 0.07–0.30; p<0.001) were protective factors. The orthodontist's experience of up to 10 years (OR:0.12; 95%CI:0.04–0.36; p<0.001), up to 40 years (OR:0.22; 95%CI:0.08–0.60; p=0.003), along with training through courses or internships (OR:0.08; 95%CI:0.04–0.18; p<0.001) or a second specialty (OR:0.37; 95%CI:0.15–0.93; p=0.035), also acted as protective factors. The proposed model has a moderately good fit (p=0.400).

**Conclusions:**

Orthodontic camouflage is the most common treatment option for Class III malocclusion. The gonial angle and anterior crossbite predispose to orthognathic surgery; while occlusal stability, the presence and spacing of the lower third molar, along with greater professional experience and surgical training, are associated with a lower likelihood of indicating surgery.

**Introduction** 

Class III malocclusion includes a series of craniofacial and dental disharmonies characterized by a mesial anteroposterior discrepancy. The diagnosis and treatment represent a challenge for orthodontist due clinical problems are result of a complex combination of genetic, environmental and developmental factors [1,2]. 

The treatment approach to Class III malocclusion will depend on growth period and severity [3]. In early stages of growth, orthopedic approach is generally aimed at achieving maxillary advancement. In adult patients, treatment depends basically on severity [4]. For example, a clinical case with slight Class III malocclusion and an acceptable facial profile can be managed by orthodontic camouflage; while in more severe cases including an unfavorable profile, can only achieve treatment goals through orthognathic surgery usually preceded a phase of orthodontic treatment [2,5]. 

In camouflage orthodontic treatment, dentoalveolar compensation is usually achieved by proinclination of the maxillary incisors, retrusion of the mandibular incisors and downward and backward rotation of the mandible. This helps to underlying skeletal problem less obvious, while allowing for an improvement in occlusion, function and esthetics [6]. However, the ultimate goals of treatment with respect to skeletal and facial appearance remain largely compromised [5,7].

In class III malocclusions with moderate to severe skeletal discrepancies, the usually recommended treatment option includes orthognathic surgery [3,5]. The orthosurgical treatment path is opposite of orthodontic camouflage because preexisting dental compensations must be eliminated and orthognathic surgery must be allowed to correct the skeletal components, seeking a functional occlusion and a noticeable improvement in facial appearance [1,4,8]. Despite the acceptable success rate, orthognathic surgery has complications that include problems such as intraoperative accidents, postoperative infection and treatment relapse [9]. 

The decision between the two interventional approaches is a challenge for the orthodontist because the treatment outcome will largely depend on an evaluation that includes anatomical and esthetic criteria [10]. While hard tissue variations are more predictable, soft tissue changes are more difficult to predict because they depend on hard tissue changes but do not necessarily correlate [11]. Furthermore, it should be taken into account that treatment routes are divergent and depending on the phase of each therapeutic approach there could be irreversible consequences, such as tooth extractions [12]. This has led orthodontists to base their treatment indication on previous training or direct or indirect experience in the management of similar cases in a valid attempt to simplify a number of variables that may influence a treatment decision [12,13].

Therefore, the aim of present study was to identify whether orthodontist factors such as professional experience, training and demographic characteristics, together with patient expectations, dental, osseous, periodontal and occlusal stability characteristics may be associated with frequency of indication for orthodontic camouflage or orthognathic surgery in patients with Class III skeletal malocclusion without growth. 

## Materials and Methods

This study followed a cross-sectional observational design, used a virtual survey as a data collection technique, and was approved by a university research ethics committee with resolution number 149-2023-USAT-FMED. The population consisted of 1134 orthodontists registered with the Peruvian Dental College.

Orthodontists registered until July 2023 and who treated at least five patients with skeletal class III malocclusion without growth in last five years were included. Orthodontists who could not be located or contacted through social networks or public profiles, who did not wish to participate or did not respond to invitation, were excluded. An elimination criterion was used to remove orthodontists who started the study but did not submit responses for any reason.

A minimum sample size of 271 orthodontists was determined using the formula for a proportion in a finite population with values N=1134, 1-α=95%, *P=*37%, E=5%; however, recruitment process allowed an adequate sample size of 287. Simple random sampling was used, with a list of specialists published on website of the Peruvian Dental College serving as sampling frame.

The identification and contact with the orthodontists was through social networks with free public access such as Facebook®, Instagram®, Twitter®, TikTok® and LinkedIn® to obtain personal and professional data. To facilitate recruitment, a public profile was implemented within each social network with referential information regarding to study, participant was added as a contact, the purpose of study was explained and selection criteria were verified. Once participation was accepted, a telephone number with access to messaging applications and/or an active e-mail address was requested to send informed consent and survey.

An inventory of 16 dichotomous and polytomous response questions was used, validated by three orthodontists and two maxillofacial surgeons with extensive experience in orthognathic surgery. The internal consistency was evaluated in 30 orthodontists to obtain a KR-20 value of 0.820. The instrument contains three sections: First includes the presentation of study and informed consent; second records demographic data and professional characteristics; third includes questions about orthodontic practice and orthognathic surgery. Table 1 describes questions and response alternatives used in survey.

The instrument was digitized in Survey Monkey® software, links were generated using codes and sent to each participant. The orthodontists responded immediately or deferred. In case of delayed responses, reminders were made every five days for up to three attempts. If there was no response, it was replaced by another orthodontist from the sample frame by probabilistic methods. A response rate of 80% was achieved with respect to the total number of surveys sent.

*Statistical analysis*

Responses were entered into a Microsoft Excel v2019 spreadsheet (Microsoft, Redmond, WA, USA) using alphanumeric codes to maintain blindness during processing. Tabulated data were exported to SPSS statistical software version 25.0 (Statistical Package for Social Sciences, SPSS Inc., Chicago, IL, USA) for univariate, bivariate and multivariate analysis. The frequency of treatment approach indication, personal and professional characteristics were represented by percentages and pie charts. Pearson's chi-square test was used to associate the indication for orthodontic camouflage or orthognathic surgery with the professional and patient variables. To evaluate the interaction of independent variables, the backward stepwise method was used to generate a binomial logistic regression model to explain the indication of treatment approach, including odds ratio (OR), beta coefficients and R² of the model; with a significance level of 5%.

## Results

This study was conducted with a sample of 287 participants, of whom 60.98% were men and 39.02% were women; the majority were orthodontists aged 41 or more years, followed by those aged 31 to 40 years old and a minority represented by the 20-30 years old. The survey showed that 37.28% of orthodontists reported between 1 and 5 years of experience, 50.17% up to 10 years, and 12.54% up to 40 years. Regarding training in orthognathic surgery, 11.15% reported having completed a course or internship, 16.03% a diploma course, 25.78% a second speciality, while 47.04% had no training in this field (Table 2). Regarding the treatment approach for a class III patient without growth, 36.59% of orthodontists preferred to choose the surgical option, while 63.41% opted for orthodontic camouflage (Figure 1).

Table 3 shows the frequency with which various factors are considered important in the decision between orthodontic camouflage and orthognathic surgery. In general, most of the professionals surveyed identified both clinical variables and patient expectations as relevant aspects, with statistically significant differences in all cases (*p<*0.001). The factors that received the highest consensus were patient expectations regarding changes in their facial profile (99.30%), cephalometric measurements related to the inclination or position of the incisors (99.30%) and the type of facial profile of the patient (98.95%). These were followed by the anteroposterior discrepancy (98.26%), the gingival biotype and the vestibular bone table of the anterior teeth (98.26%), occlusal stability (96.86%) and the dentoalveolar discrepancy (95.12%). The presence of anterior crossbite (95.82%) and anterior open bite (83.97%) were also considered relevant. Finally, the presence of a correctly erupted lower third molar and the space it provided was the factor with the lowest percentage of affirmative responses (72.82%).

Several factors showed a statistically significant association with the decision to indicate orthognathic surgery (*p<*0.05), as reported in Table 4. Measurements related to the gonial angle were identified as a significant risk factor for orthognathic surgery (OR: 5.36; 95% CI: 1.53*-*18.72; *p=*0.009); the consideration of the gonial angle measurement increases the likelihood of choosing this treatment by more than five times. Likewise, anterior crossbite is a significant risk factor for orthognathic surgery (OR: 49.14; 95% CI: 2.61*-*92.59; *p=*0.009); consideration of crossbite represented the greatest probability of choosing this treatment by more than forty times. 

Conversely, occlusal stability (OR: 0.01; 95% CI: 0.00*-*0.29; *p=*0.009) and the position and space of the third molar (OR: 0.14; 95% CI: 0.07*-*0.30; *p<*0.001) act as protective factors; the presence of occlusal stability and adequate space for the third molar significantly decreases the likelihood of orthognathic surgery being indicated. With regard to professional variables, having up to 10 years (OR: 0.12; 95% CI: 0.04*-*0.36; *p<*0.001) or up to 40 years of experience (OR: 0.22; 95% CI: 0.08*-*0.60; *p=*0.003) were associated with a lower probability of indicating surgery, acting as protective factors against the surgical decision. Similarly, training in orthognathic surgery through a course or internship (OR: 0.08; 95% CI: 0.04*-*0.18; *p<*0.001) or a second speciality (OR: 0.37; 95% CI: 0.15*-*0.93; *p=*0.035) also acted as protective factors.

In contrast, the anteroposterior skeletal discrepancy, the inclination or position of incisors, the dentoalveolar discrepancy, the anterior open bite, the gingival biotype and vestibular bone table, the type of facial profile and the patient's expectations regarding changes in facial profile, did not show a statistically significant association in the model (*p>*0.05). The proposed model fits moderately well to the observed data (*p=*0.400) and explains approximately 48.4% of the variance with respect to the decision to indicate orthognathic surgery.

## Discussion

The present study evaluated the factors associated with the indication of orthodontic camouflage or orthognathic surgery in skeletal Class III malocclusions without growth by orthodontists. This study found that orthodontists who decided to indicated orthodontic camouflage were almost twice as numerous as those who frequently chose orthognathic surgery as a treatment option; where almost half of them had no additional training in orthognathic surgery, regardless of the training they received in an orthodontic specialization program. Kauser *et al*. reported that there is a similarity in preference between orthodontic treatment and orthognathic surgery; however, unlike the present study, it is not specified how many of these professionals were orthodontists [14]. This similarity in distribution of responses between the two types of intervention was also found by Voon *et al*. when orthodontists evaluated the characteristics of a Class III malocclusion in a series of cases; although there was a slight tendency to prefer orthodontic camouflage regardless of the severity of the malocclusion, this decision was not affected by professional experience [5]. There is limited information regarding the factors that may explain the decision to indicate a treatment approach for patients with Class III malocclusions without growth [6].

All factors evaluated in this study were considered significantly important in the decision to choose a treatment approach. Patient expectations regarding profile changes and incisor-related measurements were the factors with the greatest agreement on their importance, while the presence of the third molar and the space it provides was the factor with the least consensus. This greater concordance could be due to the fact that orthodontic camouflage generally modifies the inclination of the incisors to conceal the basal discrepancy, but without achieving a correction in the skeletal base. This implies that changes in the facial profile are mostly limited and will depend on the degree of dental compensation, avoiding occlusal and periodontal instability [10,15]. In this sense, patient expectations are shown to be a determining factor when those expectations include substantial changes in the profile such as a reduction in facial concavity or a significant improvement in the lower third, where it is evident that orthodontic camouflage may be insufficient with the risk of generating post-treatment dissatisfaction that orthodontists wish to avoid [16]. In contrast, variables such as the space for the lower third molar are more controversial. In fact, this space can represent a therapeutic resource to avoid extractions and redistribute the mandibular arch length, improving orthodontic camouflage [17]. However, cone beam computed tomography studies show that the mandibular retromolar space is not constant, and the amount of space distal to the second molar varies significantly depending on different skeletal relationships, which influences the eruption of the third molar and the possibility of distalization or camouflage [18].

In this study, both the gonial angle and anterior crossbite emerged as relevant clinical variables that could influence an orthodontist's decision toward an orthosurgical approach, especially in patients without growth. The option of orthodontic camouflage is limited because an increased gonial angle is generally associated with a hyperdivergent pattern and posterior mandibular rotation, increasing the likelihood of surgical intervention [19]. Furthermore, evidence suggests that the gonial angle is a reliable indicator of mandibular growth, the severity of skeletal deformity, and the prognosis for surgical stability [12,20]. Studies using three-dimensional analysis in patients undergoing orthognathic surgery have shown that gonial morphology can be associated with the position and rotation of the proximal segment after sagittal osteotomies. In fact, variations in mandibular contour such as the gonial angle, are crucial for facial aesthetics and surgical planning, influencing the projection of the lower third of the face and the soft tissue response [21,22]. 

On the other hand, studies have indicated that although there are orthodontic camouflage alternatives in mild or moderate cases, orthognathic surgery continues to be the treatment of choice in Class III malocclusions with significant skeletal involvement, especially when there is an anterior crossbite associated with complex vertical or sagittal alterations [5,12,23]. Anterior crossbite has functional implications including alterations in anterior guidance, joint overload, and muscular adaptations [24]. This can influence the decision to opt for surgical treatment when the orthodontist suspects that dysfunctions cannot be resolved through orthodontic camouflage. Although some studies report that orthodontic treatments can improve joint parameters in early stages, evidence also suggests limitations in their effectiveness in patients with no growth potential [25].

On the other hand, the results show that a specialist’s years of experience may influence the decision to recommend surgery. In fact, orthodontists with greater clinical experience have had the opportunity to observe that orthognathic surgery is not without significant risks and complications; consequently, this experience would lead specialists to set a higher threshold for recommending surgery, prioritizing other less invasive alternatives whenever feasible [26]. Similarly, greater experience goes hand in hand with more formal training. This study also showed that orthodontists trained in orthognathic surgery are less likely to recommend the surgical procedure. Although it may seem contradictory, the trained orthodontist can better interpret cephalometric and facial analyses, recognize viable dentoalveolar compensations, and avoid classifying borderline Class III patients as surgical cases [27].

The results also show that the third molar and the space it provides in the lower jaw are another protective factor against the need for orthognathic surgery, acting as an indirect indicator of greater mandibular arch length. A greater arch length would imply a greater possibility of molar distalization or space redistribution, which would assist the orthodontist in cases of orthodontic camouflage without forcing the limits of the cortical bone [7,17,28]. Similarly, a stable occlusion was also a factor that decreased the likelihood of indicating orthognathic surgery. In fact, a malocclusion that includes dental arches with some coordination and acceptable posterior intercuspation could favor compensation for mild or moderate skeletal discrepancies [29]. If a stable occlusion can be achieved, even as a result of compensation, the patient with a Class III malocclusion can maintain a functional outcome without requiring surgery. This is because a stable occlusion implies a functional neuromuscular balance that could represent an advantage for orthodontists in terms of the risk of instability or relapse [30].

Limitations include the diversity of clinical scenarios and variables that could influence the orthodontist's decision, which are not necessarily represented in this survey. The impossibility of conducting random sampling limits the representativeness of the results, and cultural differences in different geographic areas could restrict generalization beyond the target population. The study used a self-administered survey that did not include triangulation with any objective clinical records, which could generate a risk of social desirability bias in the responses and recall bias. Considering the limited published evidence on the rationale behind clinical decisions in patients with Class III malocclusion, prospective observational cohorts with photographic and radiographic images, random comparisons of outcomes with both treatment approaches, and qualitative interviews could be included to explore in greater depth the patient's role in justifying the orthodontist's decision-making.

## Conclusions

More than half of the orthodontists surveyed significantly opted for orthodontic camouflage in the treatment of patients with non-growth class III malocclusion, compared to specialists who chose orthognathic surgery. The majority of orthodontists considered all the variables evaluated to be influential in the decision between the two approaches, with a greatest consensus in expectations of changes in facial profile, incisor inclination or position, and patient's facial profile type. 

When considering orthognathic surgery, cephalometric measurements of the gonial angle and the extent of the anterior crossbite represent significant risk factors. Meanwhile, the patient’s occlusal stability, along with the presence and space provided by the lower third molar, were characteristics that protected against the decision to recommend surgery. Likewise, characteristics of the orthodontist, such as greater clinical experience and training in orthognathic surgery through courses, internships, or specializations, were factors associated with a lower probability of recommending surgery, within a model that moderately well explains the interaction of variables in the decision to recommend orthodontic treatment or orthognathic surgery.

## Figures and Tables

**Table 1:** Table 1. Inventory type instrument used in survey.

**Question**	**Response**
Demographic data	
What is your Gender?	Male/Female
Which age group do you belong to?	20-30/ 31-40/More 41 years
Professional characteristics	
How many years of professional experience as a specialist?	1-5/ Up to 10/Up to 40 years
What is your training in orthognathic surgery?	Course or internship/Diploma/ Specialization/None
Regarding skeletal class III malocclusion	
Which treatment approach do you generally indicate for a patient with a Class III skeletal malocclusion without growth?	Orthodontic camouflage/Orthognathic surgery
Do you consider that type of facial profile of the patient is important factor for indication of orthodontic camouflage or orthognathic surgery?	Yes/ No
Do you consider that cephalometric measurements related to patient’s incisors inclination/position are important factors to indicate orthodontic camouflage or orthognathic surgery?	Yes/ No
Do you consider that cephalometric measurements related to patient's gonial angle are important factors to indicate orthodontic camouflage or orthognathic surgery?	Yes/ No
Do you consider that cephalometric measurements related to patient's anteroposterior discrepancy are important factors to indicate orthodontic camouflage or orthognathic surgery?	Yes/ No
Do you consider that presence of correctly erupted lower third molar and space provided by third molar are important factors to indicate orthodontic camouflage or orthognathic surgery?	Yes/ No
Do you consider that patient's dentoalveolar discrepancy is important factor for indication of orthodontic camouflage or orthognathic surgery?	Yes/ No
Do you consider that presence of anterior crossbite of the patient is important factor for indication of orthodontic camouflage or orthognathic surgery?	Yes/ No
Do you consider that presence of a patient's anterior open bite is important factor for indication of orthodontic camouflage or orthognathic surgery?	Yes/ No
Do you consider that gingival biotype and vestibular bone table of patient's anterior teeth are important factors to indicate orthodontic camouflage or orthognathic surgery?	Yes/ No
Do you consider that occlusal stability of patient at end of the treatment is important factor for indication of orthodontic camouflage or orthognathic surgery?	Yes/ No
Do you consider that patient's expectation regarding changes in facial profile is important factor for indication of orthodontic camouflage or orthognathic surgery?	Yes/ No

**Table 2:** Table 2. Characteristics of orthodontists in the study.

**Characteristics**		**n**	**%**
Age	20 to 30 years	10	3.48
	31 to 40 years	111	38.68
	More 41 years	166	57.84
Gender	Female	112	39.02
	Male	175	60.98
Professional experience as orthodontist	1 to 5 years	107	37.28
	Up to 10 years	144	50.17
	Up to 40 years	36	12.54
Training in orthognathic surgery	None	135	47.04
	Course or internship	32	11.15
	Diploma	46	16.03
	Specialization	74	25.78
Total		287	100.00

Categorical data expressed as absolute numbers (n) and percentages (%).

**Table 3:** Table 3. Factors considered important for the decision of orthodontic camouflage or orthognathic surgery.

**Variable**	**Response**	**n**	**%**	*p-value*
The patient's facial profile type	Yes	284	98.95	<0.001
	No	3	1.05	
The patient's expectations regarding changes in facial profile	Yes	285	99.30	<0.001
	No	2	0.70	
Cephalometric measurements related to the inclination/position of incisors	Yes	285	99.30	<0.001
	No	2	0.70	
Cephalometric measurements related to the gonial angle	Yes	254	88.50	<0.001
	No	33	11.50	
Cephalometric measurements of the anteroposterior discrepancy	Yes	282	98.26	<0.001
	No	5	1.74	
The presence of the correctly erupted lower third molar and the space it provides	Yes	209	72.82	<0.001
	No	78	27.18	
Dentoalveolar discrepancy	Yes	273	95.12	<0.001
	No	14	4.88	
The patient's occlusal stability	Yes	278	96.86	<0.001
	No	9	3.14	
The presence of anterior crossbite	Yes	275	95.82	<0.001
	No	12	4.18	
The presence of anterior open bite	Yes	241	83.97	<0.001
	No	46	16.03	
The gingival biotype and vestibular bone table of the anterior teeth	Yes	282	98.26	<0.001
	No	5	1.74	
Total		287	100.00	

Categorical data expressed as absolute numbers (n) and percentages (%). p-value: significance level ≤0.05. Z-test.

**Table 4:** Table 4. Logistic regression analysis on the factors considered important for the decision of orthognathic surgery.

**Variables**		**Coefficient**	*p-* **value**	**OR**	**95% CI**	
					**Lower**	**Upper**
Age	20 to 30 years	NE	0.717	NE	NE	NE
	31 to 40 years	0.58	0.614	1.79	0.19	17.16
	More 41 years	0.26	0.468	1.30	0.64	2.66
Professional experience as orthodontist	1 to 5 years	NE	0.001	NE	NE	NE
	Up to 10 years	-2.16	<0.001	0.12	0.04	0.36
	Up to 40 years	-1.53	0.003	0.22	0.08	0.60
Training in orthognathic surgery	None	NE	0.000	NE	NE	NE
	Course or internship	-2.51	<0.001	0.08	0.04	0.18
	Diploma	-0.57	0.293	0.57	0.20	1.63
	Specialization	-0.99	0.035	0.37	0.15	0.93
Gender (Reference: Female)		0.31	0.359	1.37	0.70	2.66
Facial profile type (Reference: Yes)		19.79	0.999	OV	0.00	0.00
Expectations regarding changes in facial profile (Reference: Yes)		1.40	0.353	4.06	0.21	78.31
Inclination/position of incisors (Reference: Yes)		-28.25	0.999	0.00	0.00	0.00
Gonial angle (Reference: Yes)		1.68	0.009	5.36	1.53	18.72
Anteroposterior skeletal discrepancy (Reference: Yes)		-19.23	0.999	0.00	0.00	0.00
Position and space of third molar (Reference: Yes)		-1.96	<0.001	0.14	0.07	0.30
Dentoalveolar discrepancy (Reference: Yes)		0.30	0.773	1.35	0.18	10.07
Occlusal stability (Reference: Yes)		-4.96	0.009	0.01	0.00	0.29
Anterior crossbite (Reference: Yes)		3.89	0.009	49.14	2.61	92.59
Anterior open bite (Reference: Yes)		-0.58	0.198	0.56	0.23	1.35
Gingival biotype and vestibular bone table (Reference: Yes)		33.94	0.999	OV	0.00	0.00
Constant		-4.49	1.000	0.01	NE	NE

NE: Not evaluated; OV: Overvalued; Hosmer and Lemeshow test: Chi2, p=0.400; Cox­ and­ Snell R^2^: 0.354; Nagelkerke R^2^: 0.484. p-value: significance level ≤0.05.

**Figure 1:**
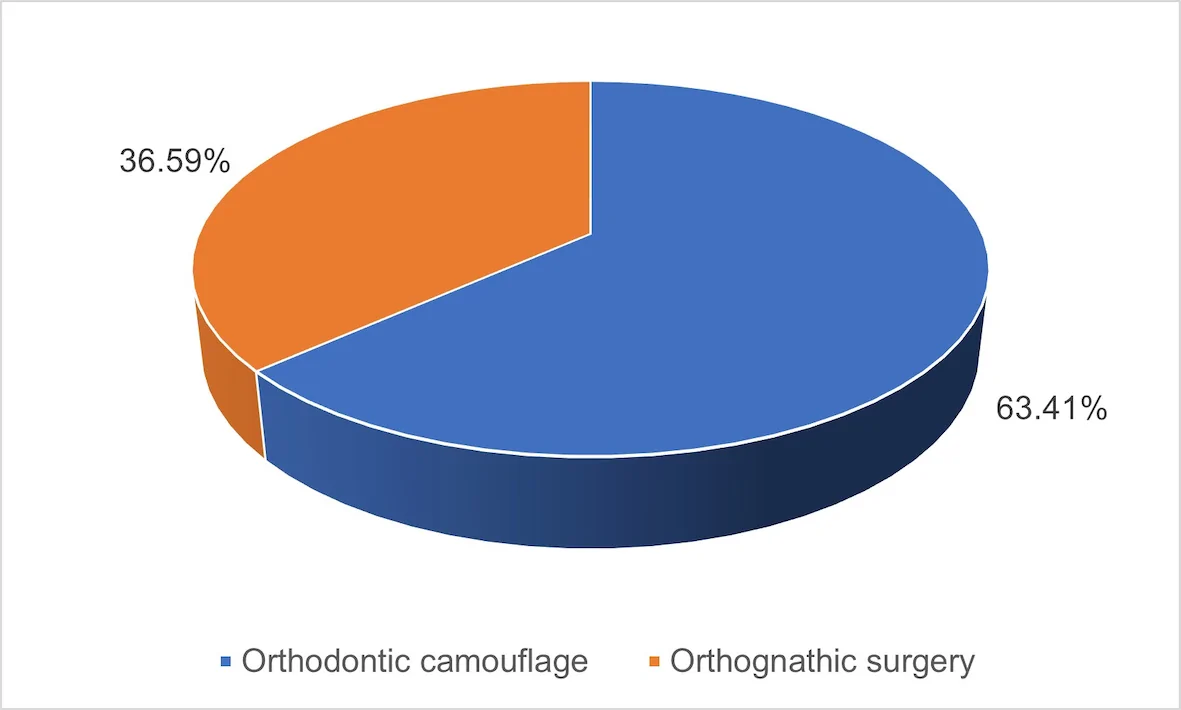
Figure 1: Treatment indication for Class III malocclusion.

## Data Availability

Declared none.
